# Structure-based neural network protein–carbohydrate interaction predictions at the residue level

**DOI:** 10.3389/fbinf.2023.1186531

**Published:** 2023-06-20

**Authors:** Samuel W. Canner, Sudhanshu Shanker, Jeffrey J. Gray

**Affiliations:** ^1^ Program in Molecular Biophysics, The Johns Hopkins University, Baltimore, MD, United States; ^2^ Department of Chemical and Biomolecular Engineering, Johns Hopkins University, Baltimore, MD, United States

**Keywords:** protein–carbohydrate binding, deep learning, site prediction, neural networks, glycan binding, oligosaccharide binding

## Abstract

Carbohydrates dynamically and transiently interact with proteins for cell–cell recognition, cellular differentiation, immune response, and many other cellular processes. Despite the molecular importance of these interactions, there are currently few reliable computational tools to predict potential carbohydrate-binding sites on any given protein. Here, we present two deep learning (DL) models named CArbohydrate–Protein interaction Site IdentiFier (CAPSIF) that predicts non-covalent carbohydrate-binding sites on proteins: (1) a 3D-UNet voxel-based neural network model (CAPSIF:V) and (2) an equivariant graph neural network model (CAPSIF:G). While both models outperform previous surrogate methods used for carbohydrate-binding site prediction, CAPSIF:V performs better than CAPSIF:G, achieving test Dice scores of 0.597 and 0.543 and test set Matthews correlation coefficients (MCCs) of 0.599 and 0.538, respectively. We further tested CAPSIF:V on AlphaFold2-predicted protein structures. CAPSIF:V performed equivalently on both experimentally determined structures and AlphaFold2-predicted structures. Finally, we demonstrate how CAPSIF models can be used in conjunction with local glycan-docking protocols, such as GlycanDock, to predict bound protein–carbohydrate structures.

## Introduction

The carbohydrate–protein handshake is the first step in many pathological and physiological processes (de Schutter and EJM van Damme, 2015; Varki et al., 2017). Pathogens attach to host cells after their lectins successfully bind to surface carbohydrates (or glycans) (Karlsson, 2001; Dyason and von Itzstein, 2010; Kato and Ishiwa, 2015; Lu and Pieters, 2019). The innate and adaptive immune systems utilize carbohydrate signatures present on cellular and subcellular surfaces to recognize and destroy foreign components (Haji-Ghassemi et al., 2015; Kappler and Hennet, 2020). Glycosaminoglycans (GAGs) bind to membrane proteins of adjacent cells for cell–cell adhesion and regulation of intracellular processes (Funderburgh, 2000; Yip et al., 2006; Angata et al., 2007). Despite the biological importance of these carbohydrate–protein interactions, few carbohydrate-specific tools leverage the vast Protein Data Bank (PDB) and recent advances in machine learning (ML) to elucidate the binding of carbohydrates at a residue level.

Knowledge of carbohydrate–protein interactions has been leveraged to develop therapeutic candidates to neutralize infections and inspire proper health function (GE et al., 2019; Lu and Pieters, 2019). One bottleneck in designing carbohydrate-mimetic drugs is obtaining residue-level interaction knowledge through methods such as structural data, mutational scanning profiles, or both (DelFernández-Alonso et al., 2012; Kieber-Emmons et al., 2014; GE et al., 2019). Furthermore, in some studies, computational tools have been used to predict docked structures, refine bound carbohydrates, or extract dynamic information (DelFernández-Alonso et al., 2012; Crawford et al., 2021; Hao et al., 2022).

Recent developments in deep learning (DL) have substantially enhanced the theoretical modeling of proteins and protein–protein interactions. For example, neural networks can design stable proteins with unique folds using graph representations (Ingraham et al., 2019; Jing et al., 2021). 3D structures can be predicted with programs such as IgFold and AlphaFold2 (AF2) (Jumper et al., 2021; Ruffolo et al., 2022a). Predicted 3D atomic coordinates can be probed to determine ligand or protein binding capabilities using neural networks, such as Kalasanty or dMaSIF (Stepniewska-Dziubinska et al., 2020; Sverrisson et al., 2021).

Recent computational studies have demonstrated new ways to explore protein–carbohydrate interactions. Our laboratory has also contributed to the advancement of this field by adding the following: (1) a shotgun scanning glycomutagenesis protocol to predict the stability and activity of protein glycovariants (Li et al., 2021) and (2) the GlycanDock algorithm to refine protein–glycoligand bound structures (Nance et al., 2021).

Recently, there have been developments in small molecule-binding site predictors. Small molecule-binding site predictors typically fall into four categories: template, geometry, energy, or ML-based (Xie and Hwang, 2015). Template-based strategies, such as 3DLigandSite (McGreig et al., 2022), search datasets for sequence and/or structurally related ligand-binding proteins to assess prospective binding sites. Geometry-based methods, such as FPocket (le Guilloux et al., 2009), search the surface of proteins for pockets and cavities. Energy-based methods, such as FTMap (Kozakov et al., 2015), use probe molecules to scan the surface of a protein to determine the energetic favorability of binding. Recently, ML techniques, such as Kalasanty (Stepniewska-Dziubinska et al., 2020), have emerged and outperformed previous classical site prediction algorithms, commonly with convolutions on a 3D voxel grid containing atomistic information (Kandel et al., 2021; Mylonas et al., 2021).

Although there are many general small molecule-binding site predictors (Kozakov et al., 2015; Stepniewska-Dziubinska et al., 2020; Evans et al., 2021), few tailored algorithms exist for the prediction of protein–carbohydrate sites. Taroni et al. (2000) analyzed carbohydrate-binding spots using the solvation potential, residue propensity, hydrophobicity, planarity, protrusion, and relatively accessible surface area to construct a function to predict carbohydrate-binding sites. Malik and Ahmad (2007) created a neural network to predict carbohydrate-binding sites using their constructed Procarb40 dataset, a collection of 40 proteins, with leave-one-out validation. Moreover, Kulharia et al. (2009) built InCa-SiteFinder to predict carbohydrate and inositol binding sites by leveraging a grid to construct an energy-based method for predicting binding sites. Tsai et al. (2012) constructed carbohydrate-binding probability density maps using an encoding of 30 protein atom types as an input to an ML algorithm. Later, Zhou, Yang, and colleagues developed two methods to predict carbohydrate-binding sites: (1) a template-based approach named SPOT-Struc (Zhao et al., 2014) and (2) a support vector machine (SVM) named SPRINT-CBH that leverages sequence-based features (Taherzadeh et al., 2016). Tsai’s method (Tsai et al., 2012) and SPOT-Struc (Zhao et al., 2014) both achieved Matthews correlation coefficients (MCC) of 0.45 on test sets of 108 and 14 proteins, respectively. The increased size of the PDB and the improvements in DL methods currently present an opportunity to train and test more broadly.

Larger protein–carbohydrate structural databases currently include UniLectin3D (Bonnardel et al., 2019) and ProCaff (Siva Shanmugam et al., 2020). UniLectin3D focuses on lectins bound to carbohydrates, containing 2,406 structures; however, it contains many redundant structures and is currently limited to 592 unique sequences. ProCaff lists 552 carbohydrate-binding protein structures and their binding affinities under various conditions; however, many structures are only available in the unbound form.

Many drug targets, from pathogen lectins to aberrant selectins, are carbohydrate-binding proteins (Ernst and Magnani, 2009; Kieber-Emmons et al., 2014; Lu and Pieters, 2019). Understanding the physiological response and determining a glycomimetic drug to neutralize the infection requires residue-level knowledge (Ernst and Magnani, 2009). Currently, DL algorithms—LectinOracle (Lundstrøm et al., 2022) and GlyNet (Carpenter et al., 2022)—predict lectin–carbohydrate binding on a protein level; however, pharmaceutical development requires residue-level information.

In this study, we develop two DL methods for residue-level carbohydrate-binding site prediction for non-covalently bound carbohydrates. The two methods have different architectures, one using voxel convolutions and the other using graph convolutions. We also present a dataset of 808 non-covalently bound nonhomologous protein chain carbohydrate structures and use it to train and test both models. We compare the performance of the models with each other and with FTMap (Kozakov et al., 2015) and Kalasanty (Stepniewska-Dziubinska et al., 2020). Then, we evaluate the performance of the models on AlphaFold2 (Jumper et al., 2021) predicted *versus* experimentally determined structures. Finally, we present a proof-of-concept pipeline to predict bound protein–carbohydrate structures.

## Results

### Dataset for carbohydrate–protein structures

To construct a method to predict carbohydrate–protein interactions, we needed a large and reliable dataset for training and testing. The dataset should contain as many nonhomologous structures as possible to avoid biasing to specific folds. By filtering the PDB (Berman, 2000), we constructed a dataset of 808 high-accuracy (<3 Å resolution), nonhomologous (30% sequence identity), and physiologically relevant experimental structures (by manually removing buffers), spanning 16 carbohydrate monomer species. When multiple copies were present in the same PDB file, we used only a single protein chain and all adjacent carbohydrate chains. In these structures, 5.2% of the protein residues contact carbohydrates (Supplementary File S1). The final dataset consists of 808 structures, which we split into 521 training structures, 125 validation structures, and 162 test structures. These structures only contain single-chain protein interactions with non-covalently bound carbohydrates.

### CAPSIF uses deep neural networks to predict carbohydrate interaction sites

We constructed convolutional neural networks (CNNs) named CArbohydrate–Protein Site IdentiFier (CAPSIF) to predict carbohydrate-binding residues from a protein structure. CNNs were initially developed for images, exploiting the spatial relationship of nearby pixels for prediction tasks. They have been applied to predict protein structure (Yang et al., 2020; Du et al., 2021; Ruffolo et al., 2022b) and small molecule-binding pockets of proteins (Stepniewska-Dziubinska et al., 2020). To predict carbohydrate-binding residues using structural information, we created two CAPSIF CNN architectures, CAPSIF:Voxel (CAPSIF:V) and CAPSIF:Graph (CAPSIF:G).

As a protein can change its side chain conformations upon binding a small molecule or carbohydrate (from *apo* to *holo*), we sought a protein representation that is robust to these and other binding-induced changes. We chose a residue-level representation, using only the Cβ positions of all residues (or Cα in glycine), as the Cβ position is frequently equivalent in both the *apo* and *holo* states (Clark et al., 2019). Both CAPSIF architectures use the following features: unbound solvent-accessible surface area (SASA) of each residue, a backbone orientation (architecture-specific), and encodings of amino acid properties, including hydrophobicity index (0–1) (Kyte and Doolittle, 1982), “aromaphilicity” index (0–1) (Hirano and Kameda, 2021), hydrogen bond donor capability (0,1), and hydrogen bond acceptor capability (0, 1) (Supplementary Table S3).

**FIGURE 1:**
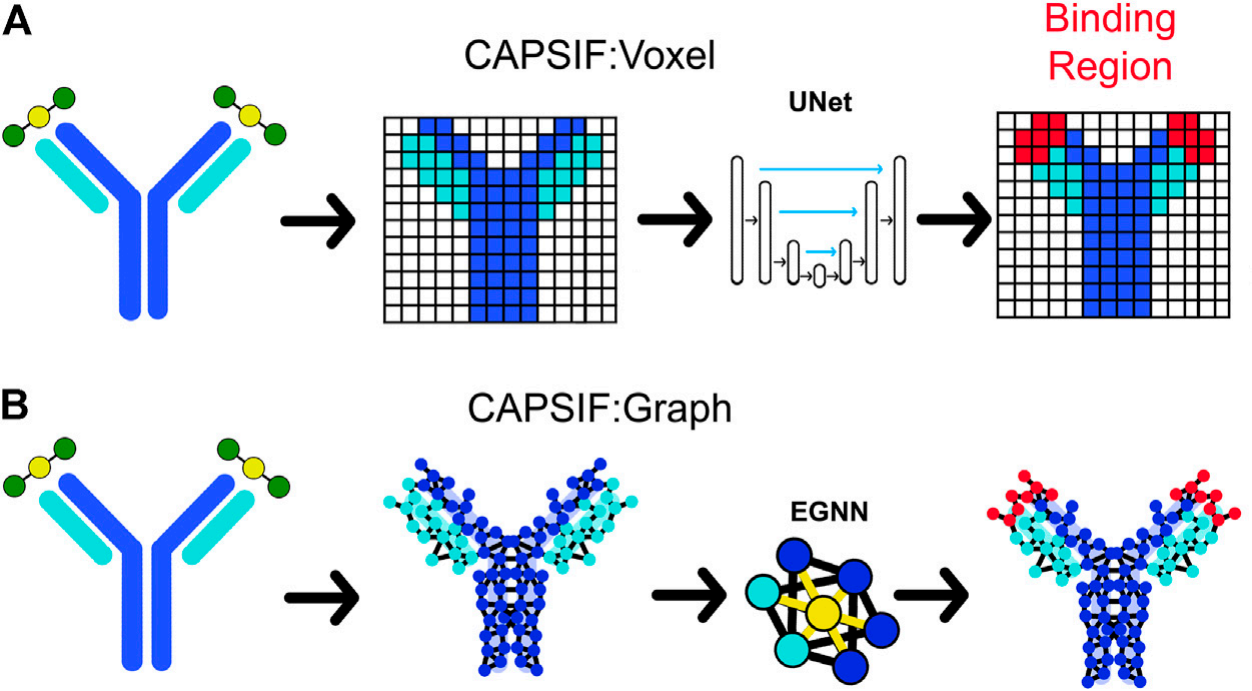
Two deep learning models that predict where proteins bind carbohydrates. **(A)** The first model (CAPSIF:V) maps the β-carbon (Cβ) coordinates into voxels, utilizes a convolutional UNet architecture, and predicts the binding residues. **(B)** The second model (CAPSIF:G) converts the Cβ coordinates into network nodes with edges for residue–residue neighbors, performs convolutions on nodes with respect to neighbors with an equivariant graph neural network (EGNN) architecture, and predicts which residues bind sugars.

The first CAPSIF architecture, CAPSIF:V, is a 3D voxelized approach to learning carbohydrate-binding pockets. CAPSIF:V uses a UNet architecture, which comprises a grid with a series of convolutions compressing and then decompressing the data to its original size with residual connections to previous layers of the same size. For each grid, we used an 8 Å^3^ voxel size where CAPSIF:V encodes each residue’s β-carbon (Cβ) into a corresponding voxel. CAPSIF:V predicts a label *P* (carbohydrate-binding residue) for each voxel on the initial grid (Figure 1A; Supplementary Figure S6).

The second architecture, CAPSIF Graph (CAPSIF:G), is an equivariant graph neural network (EGNN) (Satorras et al., 2021), with each Cβ representing a node on the graph and edges connected between all neighbor residues within 12 Å (Figure 1B). EGNNs use graph-based convolutions with message passing between connected nodes based on node features and edge features (distances) (Satorras et al., 2021). We explored many variations of these neural network architectures; Supplementary Material S1 includes data supporting our architecture and data representation choices.

The carbohydrate-binding residues comprise 5.2% of the dataset. To ameliorate the effect of data imbalance, we followed Stepniewska-Dziubinska et al. (2020) and chose the complement of the Dice similarity coefficient (*d*) as our loss function ( L=1−d ). The Dice coefficient is normalized by both the correctly and incorrectly predicted residues:

$$d=\frac{2*TP}{\left(TP+FP\right)+\left(TP+FN\right)},$$ (Eq 1)

where *TP* means true positives, *FP* false positives, and *FN* false negatives. As *d* does not depend on true negative labels, this loss function is insensitive to imbalanced datasets where the positive label is observed much less than the negative label (Stepniewska-Dziubinska et al., 2020).

### CAPSIF predicts carbohydrate-binding residues with encouraging accuracy

**FIGURE 2:**
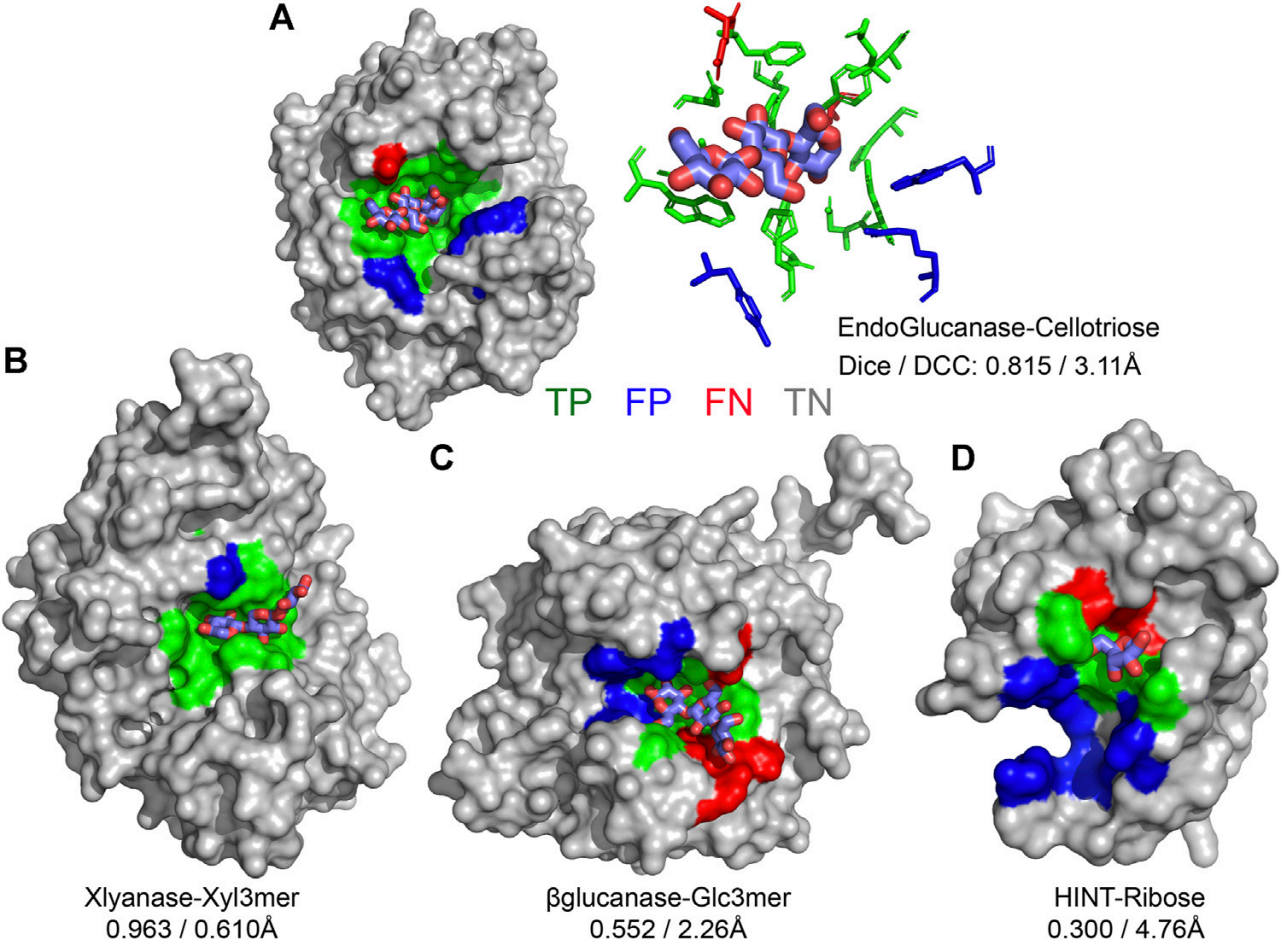
Prediction of carbohydrate-binding sites on a protein surface using CAPSIF:Voxel. **(A)** Two representations of binding residues for cellotriose bound to endoglucanase (6GL0), surface (left) and sticks (right), predicted surface representation of **(B)** xylanase bound to a xylose 3-mer (3W26), **(C)** β-glucanase bound to a glucose 3-mer (5A95), and **(D)** HINT protein bound to a ribose monomer (4RHN) predictions. True-positive residue predictions are colored green, false positives are blue, false negatives are red, true negatives are gray, and the bound carbohydrate is cyan. Dice is defined by Eq. 1, and DCC is the distance from center to center of the predicted binding regions.

CAPSIF:V and CAPSIF:G are novel architectures for predicting carbohydrate-binding residues; however, they use 512 structures to train with a substantial data imbalance. We, therefore, investigated the performance of CAPSIF on a held-out test set to determine whether the architectures accurately predict carbohydrate-binding regions despite the small amount of training data. Four representative CAPSIF:V predictions are shown in Figure 2, highlighting *TP* residue predictions (green), *FP* residues (blue), and *FN* residues (red). CAPSIF:V captures the binding pocket visually for endoglucanase (Figure 2A), xylanase (Figure 2B), and β-glucanase (Figure 2C), but it performs poorly on the HINT protein that binds ribose (Figure 2D), a five-membered ring carbohydrate that is commonly associated with nucleotides.

For comparison, we evaluated how small molecule-binding site predictors FTMap (Kozakov et al., 2015) and Kalasanty (Stepniewska-Dziubinska et al., 2020) perform for carbohydrate-binding tasks. We assessed these methods using the following metrics: the Dice coefficient (Eq. 1), distance from the center of the crystal to the center of the predicted binding location (DCC) of each independent binding site, positive predictive value (PPV), sensitivity, and Matthews correlation coefficient (MCC). Similar to the Dice coefficient, the MCC is suited for unbalanced datasets; it has been reported in previous carbohydrate-binding site studies (Tsai et al., 2012; Zhao et al., 2014; Taherzadeh et al., 2016). MCC is

$$MCC=\frac{\left(TP*TN-FP*FN\right)}{\sqrt{\left(TP+FP\right)*\left(TP+FN\right)*\left(TN+FP\right)*\left(TN+FN\right)}},$$ (Eq 2)

where *TN* means true negative. MCC ranges from −1 (worst) to +1 (best). The Dice coefficient measures the overlap of correctly predicted interacting residues with all predicted interacting residues. We define success as a Dice score greater than 0.6 or, following Stepniewska-Dziubinska et al. (2020), a DCC under 4 Å.

**FIGURE 3:**
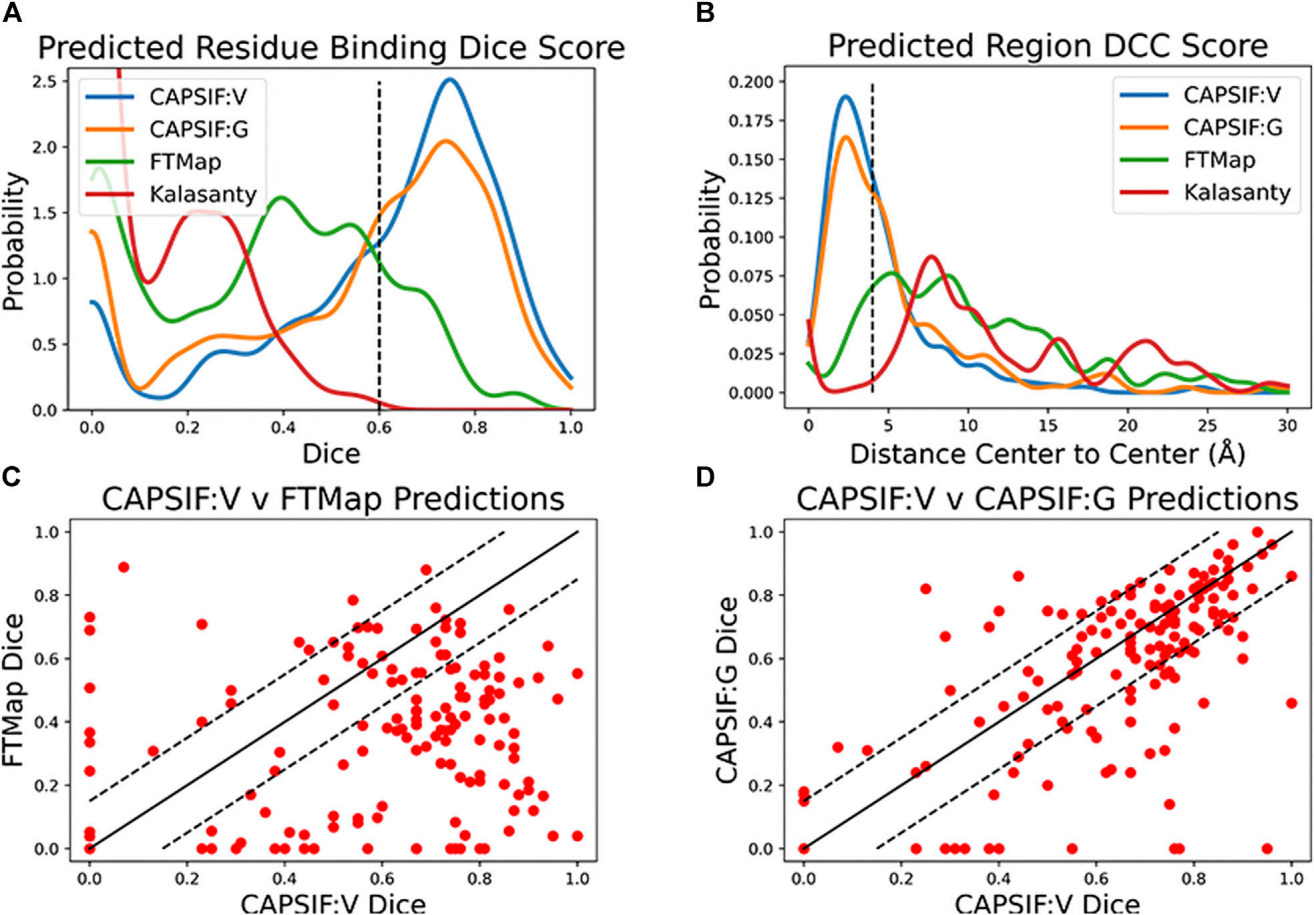
Distributions of CAPSIF:V and CAPSIF:G assessment metrics compared to FTMap (Kozakov et al., 2015) and Kalasanty (Stepniewska-Dziubinska et al., 2020). **(A)** Distribution of the Dice similarity coefficient for all methods smoothed with a Gaussian kernel density estimate (KDE, bandwidth *h* = 0.04). **(B)** Distance from center to center (DCC) of predicted to experimental carbohydrate-binding residues (smoothed with a Gaussian KDE, *h* = 0.75 Å). **(C)** Per-target comparison of CAPSIF:V to FTMap and **(D)** CAPSIF:G Dice coefficients.

On the CAPSIF test set, FTMap achieved an average Dice coefficient of 0.351 and an average DCC of 10.5 Å, and Kalasanty achieved an average Dice coefficient of 0.108 and an average DCC of 14.6 Å (Table 1). Furthermore, FTMap predicted 16.8% of test structures with greater than 0.6 Dice and 16.8% of test structures with less than 4 Å DCC, whereas Kalasanty predicted 0% of test structures with greater than 0.6 Dice and 21.4% of test structures with less than 4 Å DCC (Table 1; Figures 3A,B).

**TABLE 1:** Average metric for each method on the test set. The Dice similarity coefficient is defined by Eq. 1, PPV is positive predictive value = TP/(TP + FP), sensitivity = TP/(TP + FN), DCC is the distance from center to center of predicted *versus* experimentally determined residues and only calculated for proteins that yield predictions (coverage), and MCC is the Matthews correlation coefficient and defined by Eq. 2. Boldface indicates best performance for each metric.

Model	Dice	PPV	Sensitivity	DCC (Å)	MCC	Coverage (%)
**FTMap**	0.351	0.284	0.505	10.56	0.222	**100.0**
**Kalasanty**	0.108	0.080	0.207	14.62	−0.624	90.0
**CAPSIF:V**	**0.597**	**0.598**	**0.647**	**4.48**	**0.599**	94.4
**CAPSIF:G**	0.543	0.541	0.590	5.85	0.538	83.2

We then investigated whether our CAPSIF models, which are specifically tuned for carbohydrate binding, predict the carbohydrate-binding regions more accurately than Kalasanty and FTMap. On the held-out CAPSIF test set, CAPSIF:V achieves an average 0.597 Dice coefficient and 4.48 Å DCC metric, and CAPSIF:G achieves an average 0.543 Dice coefficient and 5.85 Å DCC metric (Table 1). Furthermore, CAPSIF:V successfully predicts 62.7% of test structures with greater than 0.6 Dice and 56.5% of test structures with less than 4 Å DCC, and CAPSIF:G successfully predicts 55.2% of test structures with less than 0.6 Dice and 46.0% of test structures with less than 4.0 Å DCC. Both CAPSIF models have a most probable prediction at 0.77 Dice and 2.5 Å DCC (Table 1; Figures 3A,B).

As CAPSIF is ML-based and FTMap is energy-based, FTMap may predict more accurately in different cases compared to CAPSIF. We compared the CAPSIF:V and FTMap Dice scores for each structure (Figure 3C). FTMap achieves significantly higher Dice coefficients (difference greater than 0.15 Dice coefficient) than CAPSIF:V in 10.9% of cases, and CAPSIF:V predicts the binding region with a significantly greater Dice coefficient than FTMap in 67.9% of cases. We also compared the computer time. On The FTMap server, FTMap requires an hour or more to predict the binding region for a single structure, whereas both CAPSIF:V and CAPSIF:G predict binding sites within seconds on a single CPU. Thus, on average, CAPSIF:V and CAPSIF:G outperform current small molecule-binding site predictors of carbohydrate binding.

Finally, we compared the CAPSIF:V and CAPSIF:G architectures. CAPSIF:V has an average Dice coefficient of 0.597 and CAPSIF:G has an average Dice coefficient of 0.543 across the test dataset (Table 1). When comparing the Dice on the test set, CAPSIF:V predicts 27.3% of structures with greater than 0.15 Dice coefficient compared to CAPSIF:G, whereas CAPSIF:G predicts 11.2% of structures with greater than 0.15 Dice coefficient compared to CAPSIF:V (Figure 3D). Thus, CAPSIF:V outperforms CAPSIF:G in carbohydrate-binding site prediction.

Carbohydrates are unique biomolecules that bind to different lectins with high specificity. Both CAPSIF architectures treat all carbohydrates agnostically, meaning that all sugar residue types are considered equivalent for predictions. Nonetheless, we compared prediction results across different sugar residue types (Supplementary File S1). CAPSIF:V performs best on glucose (Glc), galactosamine (GalN), arabinose (Ara), xylose (Xyl), ribose (Rib), and galacturonic acid (GalNAc). It predicts regions that bind neuraminic acid (Neu/Sia), fucose (Fuc), and glucuronic acid (GlcNAc) with less than an average 0.5 Dice coefficient. The weaker performance could stem from chemical differences or differences in the size of the training data. Neu and Fuc are substantially chemically distinct carbohydrates, as Neu is a 9-carbon structure and Fuc adopts an (*L*) conformation; both are sparse in our dataset. Furthermore, CAPSIF:V performs best on transport proteins, membrane proteins, and hydrolases; however, it performs weakly on viral proteins and lyases (Supplementary File S1).

### CAPSIF:Voxel, in most cases, performs similarly on AlphaFold2 structures

**FIGURE 4:**
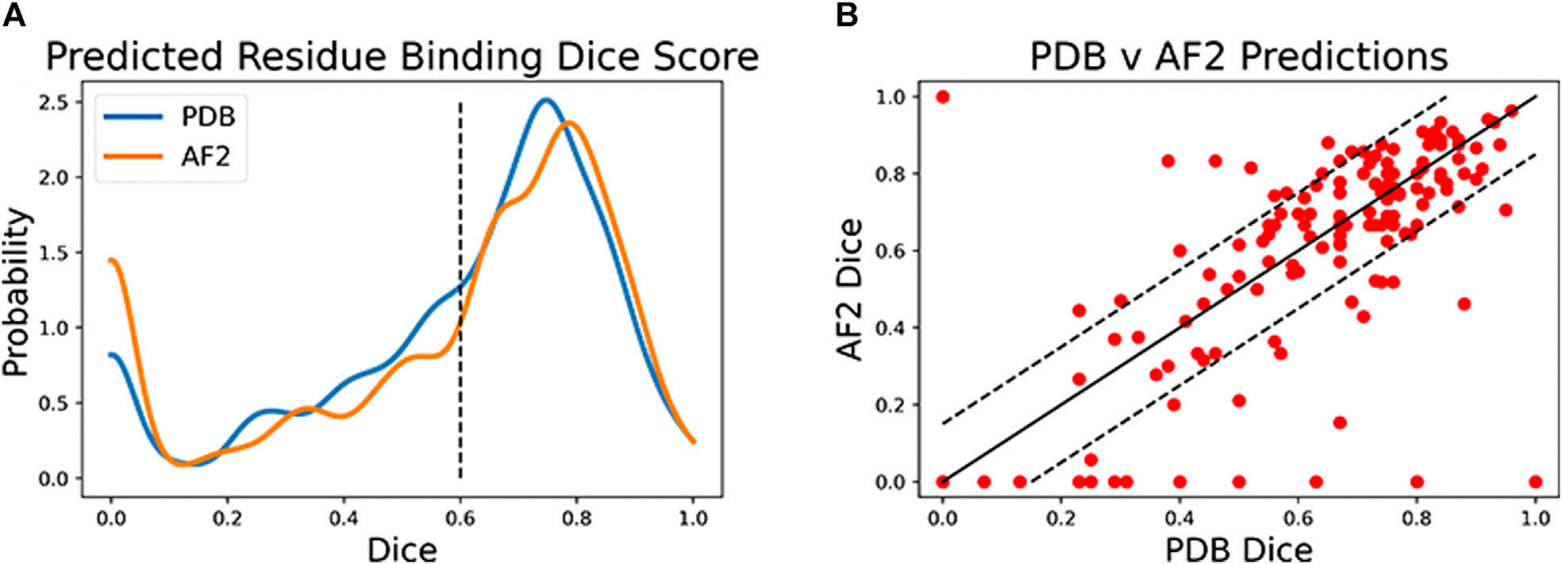
Dice coefficient assessment of CAPSIF:Voxel on PDB and AlphaFold2 (AF2) structures. **(A)** Kernel density estimate (*h* = 0.04) showing the distribution of the Dice coefficient for both methods. **(B)** Comparison of each test structure between CAPSIF:V on PDB and AF2 structures.

Both CAPSIF models were trained and tested on bound crystal structures; however, experimental protein structure determination can be expensive, even in the absence of carbohydrates. We, therefore, investigated whether CAPSIF:V could usefully predict carbohydrate-binding structures from computationally modeled structures. We reconstructed the test protein structure dataset with the Colab implementation of AlphaFold2 (AF2) (Jumper et al., 2021; Mirdita et al., 2022), predicted the carbohydrate-binding residues of the predicted structures, and evaluated the same performance metrics (Table 2). CAPSIF:V predicts the carbohydrate-binding regions with similar Dice coefficients of 0.597 and 0.586 for PDB *versus* AF2 predicted structures, respectively. Figure 4A shows that the Dice distribution is similar between PDB and AF2 structures. CAPSIF:V predicts the center of the carbohydrate-binding region more accurately on AF2 structures with a DCC of 3.8 Å, compared to 4.5 Å on crystal structures.

**TABLE 2:** Metrics for CAPSIF:Voxel inputting PDB or AF2 structures. Dice, PPV, sensitivity, DCC, MCC, and coverage defined in Table 1.

Structure	Dice	PPV	Sensitivity	DCC (Å)	MCC	Coverage (%)
PDB	0.597	0.598	0.647	4.48	0.599	94.4
AF2	0.586	0.508	0.744	3.76	0.598	85.0

Although CAPSIF:V has a lower average DCC on AF2 structures compared to experimental structures, CAPSIF:V fails to predict any sites at all on 15% of AF2 structures, whereas it fails in only 5% of PDB structures, suggesting that the signal about sugar binding is removed for some of the small backbone errors produced by AF2.

The multiple outliers where CAPSIF:V fails to predict the region of carbohydrate binding in only AF2-predicted structures are sorted in Figure 4B. CAPSIF:V predicts a Dice coefficient of at least 0.15 units higher for PDB structures in 14.9% of structures and predicts AF2 structures with a 0.15 Dice coefficient or higher for 8.7% of test structures. AF2-generated structures can be inaccurate; however, in most of the test cases, AF2 captures the structures with angstrom level accuracy and the carbohydrate-binding residues with high pLDDT confidence; unfortunately, the pLDDT confidence measure does not correlate with the CAPSIF success rate (Supplementary Figure S8).

### CAPSIF assists *ab initio* prediction of bound protein–carbohydrate structures

CAPSIF:V predicts the carbohydrate-binding site on the majority of proteins with high accuracy, suggesting that it might be used in a pipeline to predict bound protein–carbohydrate structures. As a proof-of-concept, we developed a prospective pipeline and tested it on five proteins from the GlycanDock (Nance et al., 2021) test dataset that were not included in the CAPSIF dataset.

We constructed the following rudimentary pipeline. We predicted the binding site from each unbound protein’s experimentally determined structure with CAPSIF:V and constructed the known carbohydrate with Rosetta. The carbohydrate center of mass (CoM) was then placed in the CoM of the predicted binding region and manually rotated to align with the binding region shape. Subsequently, we used the Rosetta FastRelax (Tyka et al., 2011) protocol to remove steric clashes. In addition, we used Rosetta’s standard GlycanDock (Nance et al., 2021) to predict the bound structures. To find the highest-rated bound structure, we filtered 9,500 decoys by their computed interaction energy.

**FIGURE 5:**
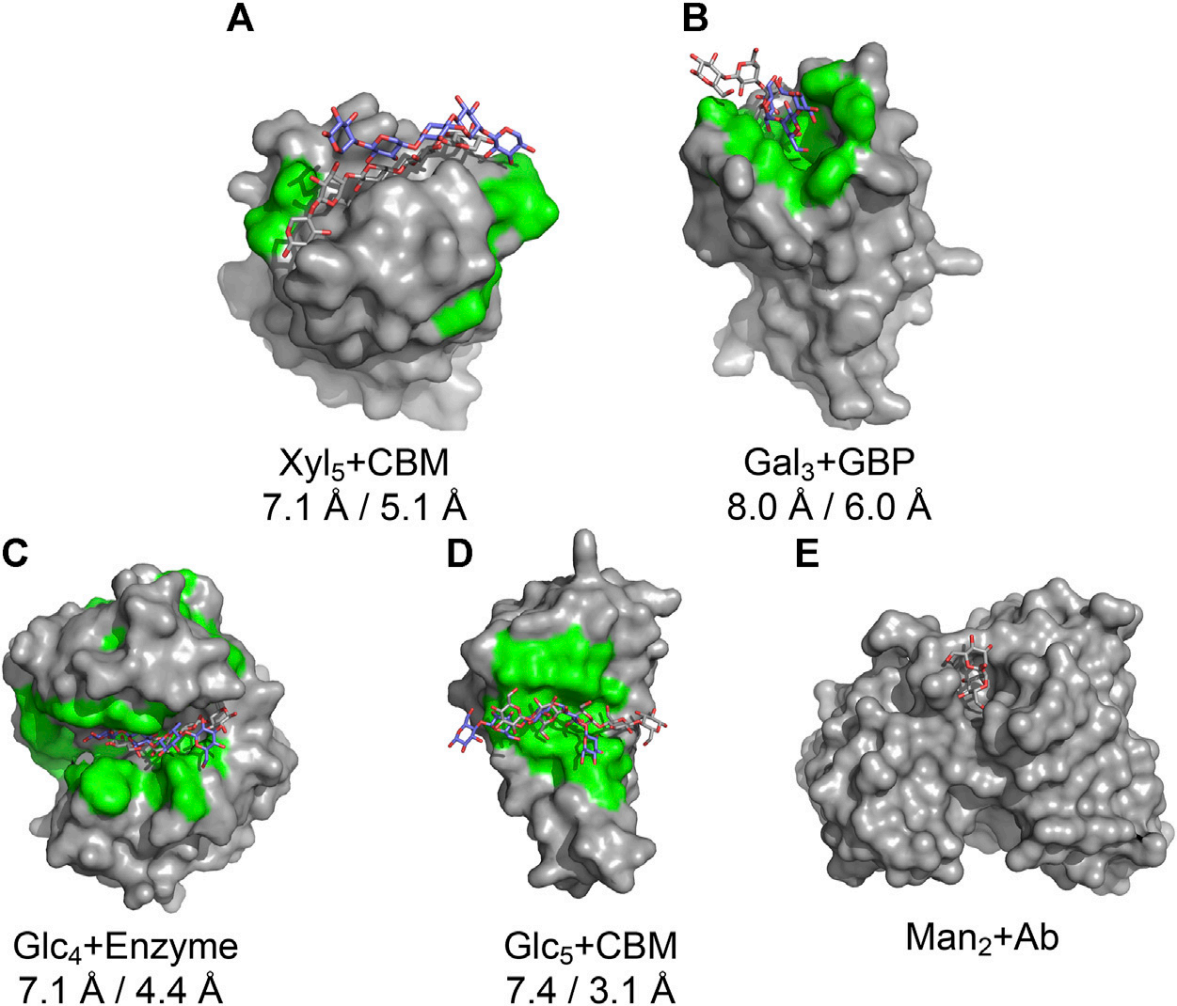
Results of the CAPSIF:V-GlycanDock pipeline. CAPSIF-predicted residues are shown in green. Wild-type unbound structures are shown in surface representation in gray, with the experimentally determined carbohydrate in gray sticks and predicted bound carbohydrate in purple sticks. RMSD of the entire ligand and RMSD of the register-adjusted ligand are shown. **(A)** A carbohydrate-binding module (CBM), 1GMM (unbound PDB)/1UXX (bound PDB), **(B)** a glycan-binding protein (GBP), 1L7L/2VXJ, **(C)** an enzyme, 1OLR/1UU6, **(D)** a CBM, 2ZEW/2ZEX, and **(E)** an antibody (Ab), 6N32/6N35 are shown.

We tested the pipeline on five experimentally solved unbound proteins: *P. aeruginosa* lectin 1, a glycan-binding protein (GBP, 1L7L), two carbohydrate-binding modules (CBMs) (viz., 1GMM and 2ZEW), a glycoside hydrolase enzyme (1OLR), and an anti-HIV-1 antibody (Ab) (6N32). Figure 5 shows structures and the root mean squared deviation (RMSD) of each predicted carbohydrate structure from the experimental structure. CAPSIF:V predicts carbohydrate-binding residues near the correct site on four of the five proteins, but it fails to predict any binding residues on the antibody (6N32). For three of the proteins, CAPSIF:V predicts the region with high accuracy. However, on 1GMM, CAPSIF:V predicts regions flanking the binding site, but it still provides a CoM similar to the actual binding region. For the carbohydrates with identified sites, the standard GlycanDock protocol was able to refine the carbohydrate structure to an RMSD of less than 8 Å for the entire ligand and less than 6 Å for register-adjusted values, where the termini were removed before calculating RMSD. The 3-mer Gal GBP (1L7L) has the worst RMSD (6 Å register adjusted, Figure 5B) likely because the *holo* conformation (2VXJ) undergoes a conformational change at the carbohydrate-binding site. Although this Ab case example failed, CAPSIF successfully predicted the carbohydrate-binding regions of 9 of the 11 Abs tested from the GlycanDock test set, which has no overlap with the CAPSIF training set. These predictions demonstrate the potential of CAPSIF to help inform experimental hypotheses or for high-throughput predictions of bound protein–carbohydrate structures.

## Discussion

We demonstrated that both CAPSIF models predict residues of proteins that bind carbohydrates with much higher accuracy than prior approaches. CAPSIF:V uses a voxelized approach and predicts 62.7% of crystal structures with a distance from the center of the predicted region to the center of the experimentally determined region (DCC) within 4 Å. CAPSIF:G performs strongly on the dataset, predicting 55.2% of crystal structures with a DCC less than 4 Å, with CAPSIF:V performing similarly or outperforming CAPSIF:G in 88.8% of cases. CAPSIF:V is robust to most errors in the protein structure of the magnitude in AF2 structures (Ångström-level) (Jumper et al., 2021): the algorithm predicts similar carbohydrate-binding residue regions independent of whether the input structure is experimentally determined or predicted by AF2. This algorithm is a substantial improvement over surrogate ligand site predictors, Kalasanty and FTMap.

Furthermore, CAPSIF outperforms previous methods specifically tuned for carbohydrate binding. CAPSIF:V achieved a 0.599 MCC, and CAPSIF:G achieved a 0.538 MCC on the test dataset. Tsai et al., 2012’s method using probability density maps achieved a 0.45 MCC on their independent test dataset of 108 proteins, SPOT-Struc achieved a 0.45 MCC on their test dataset of 14 proteins (Zhao et al., 2014), and SPRINT-CBH achieved an MCC of 0.27 MCC on their test set of 158 proteins (Taherzadeh et al., 2016). Although these datasets differ from ours, ours is a similarly constructed nonhomologous dataset of 162 structures, and CAPSIF has a markedly stronger MCC. Although CAPSIF:V performs best, we advocate for using CAPSIF:V and CAPSIF:G in tandem to predict carbohydrate-binding residues because there are numerous cases where one CAPSIF model outperforms the other.

Although CAPSIF accurately captures the protein–carbohydrate-binding interface, there are limitations. CAPSIF is carbohydrate-agnostic, so it only predicts that a protein residue will bind one of the 16 carbohydrate monomers. In other words, CAPSIF predicts the location of carbohydrate binding, but it does not predict which carbohydrate preferentially binds there. Furthermore, CAPSIF was only trained and tested on known non-covalent carbohydrate-binding proteins. Therefore, CAPSIF may not be informative on non-carbohydrate-binding proteins or proteins that bind glycoconjugates, such as ribose in nucleic acids, ATP/ADP, or GTP/GDP (Supplementary Figure S10). CAPSIF was trained on a small set of sixteen sugar residue types, and it will be most useful for non-modified sugar residues. Another limitation is that CAPSIF fails to predict any binding on about three times as many AF2-predicted structures as crystal structures. Unfortunately, CAPSIF prediction accuracy on AF2 structures is not correlated with pLDDT confidence metrics, so it is not possible to know when it will fail. Furthermore, CAPSIF was tested on AF2-predicted structures for proteins that already exist and may exist in the AF2 training set.

The scope of CAPSIF makes it well-suited for a computational pipeline. We suggest the use of DeepFRI (Gligorijević et al., 2021), a DL model that predicts protein function, to first determine whether it is a carbohydrate-binding protein. If it is a carbohydrate-binding protein, then LectinOracle (Lundstrøm et al., 2022) or GlyNet (Carpenter et al., 2022) can be used to predict which carbohydrates bind the protein. CAPSIF can then predict binding locations, either from an experimental structure or AF2-generated structures, and then GlycanDock (Nance et al., 2021) can predict a docked protein–carbohydrate structure.

We tested part of this pipeline by predicting the binding region using CAPSIF:V and docking the known carbohydrate binder to the region with GlycanDock (Nance et al., 2021). CAPSIF:V predicted binding sites on four of the five proteins. The antibody case, which failed, bound a carbohydrate at the complementary determining region (CDR) loops, split over two chains, but CAPSIF was trained only on single chain data. When the register was adjusted, each structure yielded a ligand RMSD less than 6 Å. We anticipated that a more well-tuned pipeline could yield higher accuracy structures *ab initio* from sequence only.

To our knowledge, voxelized and graph-based site prediction has not been presented simultaneously before. Existing studies have used graphs to either predict binding affinity (Jones et al., 2021) or a docked structure (in coordination with diffusion techniques) (Corso et al., 2023). However, they have not been used to determine small molecule-binding regions. We tested two architectures utilizing either voxel or graph representations. We showed that CAPSIF:V outperforms CAPSIF:G, both of which use convolutions to predict the carbohydrate-binding ability of residues with the same residue representation. We can speculate about the reason by considering the differences between the approaches. CAPSIF:V discretizes the protein structure over a 3D grid, which can obscure the Cβ position by a few Ångströms, whereas CAPSIF:G uses the coordinates without any loss of spatial information. CAPSIF:V encodes the initial 1.4 M features input to a lower dimensionality of a 512-feature vector to encode the entire structure, whereas CAPSIF:G lifts the data from an *N*_res_ × 30 to a higher dimensionality of *N*_res_ × 64. CAPSIF:V has 102 M parameters, and CAPSIF:G has 236K parameters, reflecting how graph-based methods capture the spatially equivariant information in fewer parameters. One characteristic of using the voxel representation is that the grid contains voxels with the protein and the voxels outside the protein, including binding pocket cavities, whereas the graph representation only contains the protein. Voxel network reasoning over the surface pocket volume may be the key factor for improved carbohydrate-binding residue prediction.

By building on this initial screen, future studies could focus on improving the CAPSIF data representation for improved accuracy and extending these models to predict which carbohydrate monomer a residue most preferentially binds and whether the protein is a carbohydrate-binding protein. In the future, the dataset could include oligomeric structures that bind carbohydrates at the oligomeric interface. Furthermore, model performance could be improved by leveraging homologous structures with data splits across families. Although lectins are well known for carbohydrate binding, some protein families, such as G protein-coupled receptors (GPCRs) and antibodies, do not exclusively bind carbohydrates (Dingjan et al., 2015; Yang et al., 2021). Additionally, with our carbohydrate-binding site dataset, one could test small molecule-binding site predictor neural networks such as Kalasanty (Stepniewska-Dziubinska et al., 2020) or PeSTo (Krapp et al., 2023) by fine-tuning them for sugars. High-throughput methods like these could enable proteomic scale sorting of carbohydrate-binding capabilities.

## Methods

### Dataset

No dataset of nonhomologous bound protein–carbohydrate structures that leveraged the total size of the current PDB existed, so we constructed one. A simple selection of all RCSB (Berman, 2000) structures with carbohydrates gives all docked protein–carbohydrate structures but also inherently returns all glycosylated proteins, glycosylated peptides, and all protein structures that use carbohydrates as crystallization agents. We aimed to determine all true physiological protein–carbohydrate interactions; therefore, we manually removed nonspecific crystallization buffers or glycoproteins. Subsequently, we removed all proteins with a resolution over 3 Å and removed all homologous protein structures with over 30% sequence identity to remove all sequentially redundant proteins, only accounting for chain homology and not domain homology. Some structures containing sugars with modified monosaccharides and cyclic carbohydrates were unreadable in the PyRosetta (Chaudhury et al., 2010) software and, therefore, additionally removed.

The final dataset consists of 808 structures, with a split of 521 training structures, 125 validation structures, and 162 test structures. Each structure has one or more of the following carbohydrate monomers: glucose (Glc), glucosamine (GlcNAc), glucuronic acid (GlcA), fucose (Fuc), mannose (Man), mannosamine (ManNAc), galactose (Gal), galactosamine (GalNAc), galacturonic acid (GalA), neuraminic acid (Neu)/sialic acid (Sia), arabinose (Ara), xylose (Xyl), ribose, rhamnose (Rha), abequose (Abe), and fructose (Fru). We split the training, validation, and test sets pseudo-randomly to ensure an equal representation of all carbohydrate species in each split. The numbers of each monomer per structure and the Dice coefficient for each carbohydrate monomer type and each protein family in the test set from CAPSIF:V are included in Supplementary File S1. For all the following work, we defined a carbohydrate-interacting residue as residues with any heavy atom within 4.2 Å of a carbohydrate-heavy atom.

### CAPSIF:V data processing

CNNs are not rotation invariant, and so data augmentation by rotations improves their performance (Villar et al., 2021). Therefore, we augmented the input data for CAPSIF:V during training to overcome the rotational variance. Each time a structure was used in training, it was rotated in Cartesian space by a random angle in {−180°,180°} around an axis defined by a randomly chosen residue’s location and the protein center of mass. With the random rotation for each epoch, the network learned approximately 1,000 different orientations of each structure in the dataset. If the protein was too large for the grid size, it was split into separate grids and run separately (approximately 22% of the training points).

### Neural network architectures

#### Features

Due to the small dataset size of 808 structures, we chose residue-level representations instead of atomistic ones. We assigned all residue information to the Cβ atom of each residue because the position of the Cβ is similar in the *apo* and *holo* states (Clark et al., 2019). The features are listed in Table 3. The SASA, hydrophobicity, and H bond donor/acceptor indices were calculated using PyRosetta (Chaudhury et al., 2010), and aromaphilicity was indexed by Hirano and Kameda (2021).

**TABLE 3:** List of features and the associated encoding size used for both CAPSIF models.

Feature type	Encoding size
Amino acid (one-hot)	20
SASA	1
Hydrophobicity	1
Aromaphilicity	1
H bond donor/acceptor	2
Orientation (voxel only)	3
Torsion (graph only)	4

#### CAPSIF:Voxel

CAPSIF:V utilizes a UNet architecture, encoding and decoding the input structure to predict carbohydrate-binding residues with residual connections. CAPSIF:V inputs a grid of 36 × 36 × 36 voxels, with each voxel representing 2 Å × 2 Å × 2 Å. We input a tensor of sizes (28, 36, 36, 36), with the 28 features from Table 3, where orientation is the normalized components of the Cα to Cβ bond vector. All voxels without a Cβ within are input as zero vectors.

**FIGURE 6:**
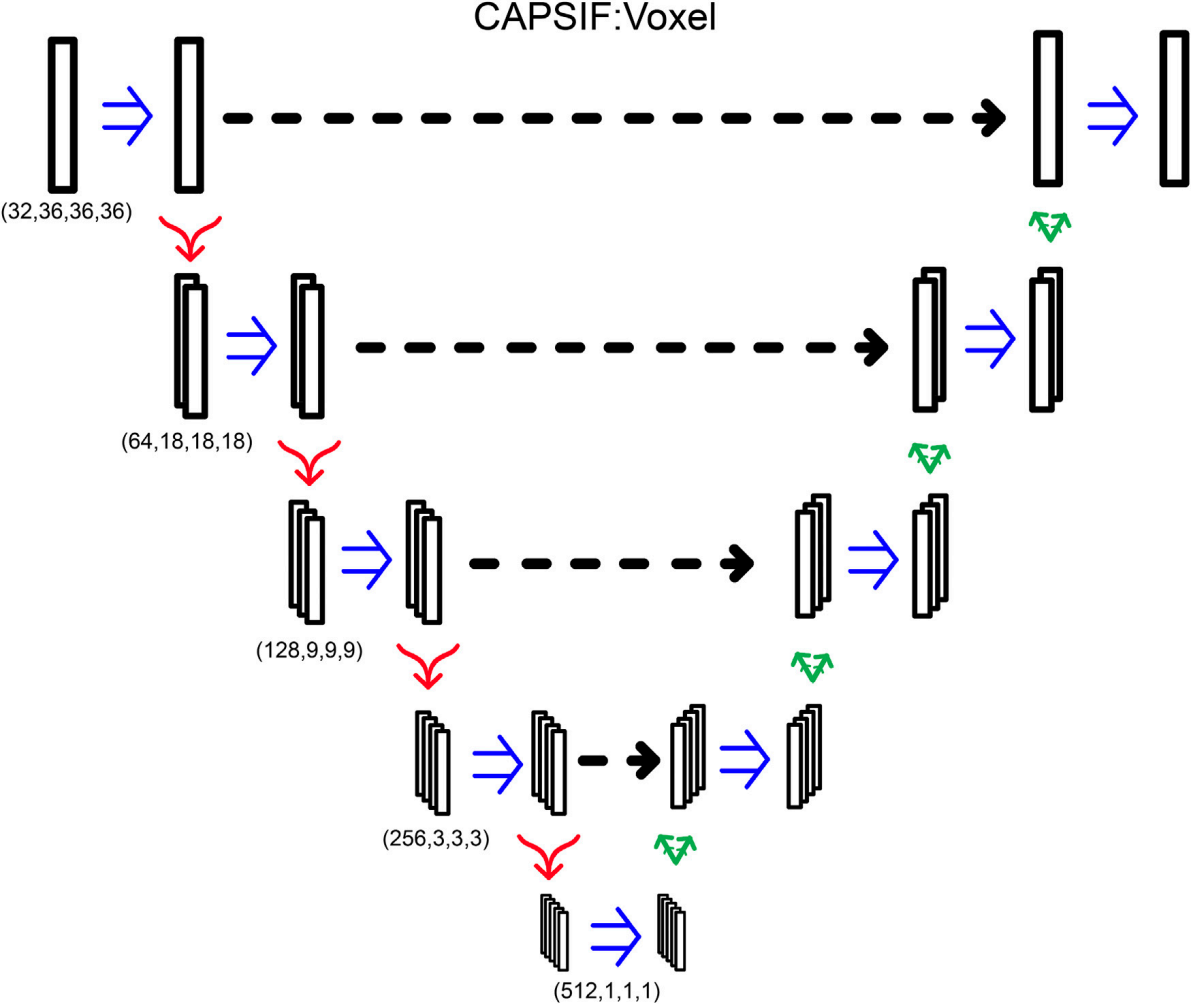
CAPSIF:V architecture. Blue arrows indicate a double convolution, red arrows indicate an encoding layer, and green arrows indicate a decoding layer.

CAPSIF:V contains an embedding layer and nine convolutional blocks where four blocks encode the structure, one block forms the bottleneck, and four blocks decode the structural information. The embedding layer lifts the 28-channel input into a 32-dimension space. Each block has a double convolution, performing the following methods twice: 3D convolution, with the same number of input channels as the number of output channels, (5 × 5 × 5) kernel with a stride of 1 and padding of 2, a batch normalization layer, and a rectified linear unit (ReLU) activation function. In addition, each encoding block has a MaxPooling layer to double the size of the channels (32, 64, 128, 256, 512) while reducing the 3D cubic voxel numbers (36, 18, 9, 3, 1). Each decoding block first concatenates the results of the encoding layer of the same size and then performs a double convolution and a 3D-transposed convolution operator, reducing the number of channels (256, 128, 64, 32) while increasing the 3D cubic voxel numbers (3, 9, 18, 36) After the nine blocks, there is a single convolutional layer condensing the input channels (32) into a single output channel, which is then followed by a sigmoid activation function to output the probability that the voxel contains a residue that binds a sugar (Figure 6). CAPSIF:V contains 102,676,001 parameters.

CAPSIF:V was trained for 1,000 epochs with a learning rate of 10^−4^ and batch size of 20 grids using the Adam (Kingma and Ba, 2015) optimizer with the loss function L=1−d , where *d* is defined by Eq. 1.

In optimizing CAPSIF:V, we explored several model variations. We tested various combinations of 3 × 3 × 3, 5 × 5 × 5, and 7 × 7 × 7 convolutional filters. We used four convolutions per layer instead of the double convolution in the primary model. Furthermore, we used larger voxel grid sizes (72 × 72 × 72 instead of 36 × 36 × 36) with another decoding/encoding layer in the UNet architecture. We also attempted different configurations of skip connections, such as UNet++ (Zhou et al., 2018). These models required slower learning rates and showed slower convergence with no improvement in prediction quality than the presented model. The best model for validation accuracy is described previously.

#### CAPSIF:Equivariant graph neural network

CAPSIF:G is an equivariant graph neural network (Satorras et al., 2021) that performs convolutions on each node (chosen as each Cα for glycine and Cβ for all others). Graph edges are connected between neighbors (defined as all other nodes within 12 Å), and the edge attribute is the distance between node Cβ atoms. In addition to the features used in CAPSIF:V, we include a torsional component in the node features as the sine and cosine of the φ and ψ angles of each residue (Table 3).

CAPSIF:G first lifts the 29-feature input node into a 64-dimension space. The 64-feature vector, alongside the edge features (distances), is then input to eight consecutive equivariant graph convolutional layers (EGCLs) (Satorras et al., 2021). Each EGCL contains an edge multilayer perceptron (MLP), a node MLP, a coordinate MLP, and an attention MLP. The edge MLP consists of two blocks of a linear layer and a rectified linear unit (ReLU) activation function. The node MLP consists of a linear layer, a ReLU activation layer, and a linear layer. The coordinate MLP contains a linear layer, a ReLU activation layer, and a linear layer. The attention MLP contains a linear layer and a sigmoid activation function. All layers input and output a 64-feature vector. Finally, CAPSIF returns the embedding to a 29-feature vector per node, adds the initial input features to the final vector, performs batch normalization, and then uses a sigmoid activation function to output a probability of carbohydrate binding of all residues. CAPSIF:G contains 236,009 parameters.

This model was trained for 1,000 epochs with a learning rate of 10^−4^ and batch size of one protein using the Adam optimizer (Kingma and Ba, 2015) with the loss function L=1−d , where *d* is defined by (Eq. 1).

In optimizing CAPSIF:G, we explored changing the number of graph convolutional layers and the latent space dimensionality. We tested the number of layers (*L* = 4,6,8,16) and used different dimensionalities of the latent space (*d* = 16,32,64). The best-performing model is described previously.

## Data Availability

The datasets and code presented in this study can be found in the following online repository: https://github.com/Graylab/CAPSIF.
